# Fluoromycobacteriophages for Rapid, Specific, and Sensitive Antibiotic Susceptibility Testing of *Mycobacterium tuberculosis*

**DOI:** 10.1371/journal.pone.0004870

**Published:** 2009-03-20

**Authors:** Mariana Piuri, William R. Jacobs, Graham F. Hatfull

**Affiliations:** 1 Department of Biological Sciences, University of Pittsburgh, Pittsburgh, Pennsylvania, United States of America; 2 Howard Hughes Medical Institute, Department of Microbiology and Immunology, Albert Einstein College of Medicine, Bronx, New York, United States of America; McGill University, Canada

## Abstract

Rapid antibiotic susceptibility testing of *Mycobacterium tuberculosis* is of paramount importance as multiple- and extensively- drug resistant strains of *M. tuberculosis* emerge and spread. We describe here a virus-based assay in which fluoromycobacteriophages are used to deliver a GFP or ZsYellow fluorescent marker gene to *M. tuberculosis*, which can then be monitored by fluorescent detection approaches including fluorescent microscopy and flow cytometry. Pre-clinical evaluations show that addition of either Rifampicin or Streptomycin at the time of phage addition obliterates fluorescence in susceptible cells but not in isogenic resistant bacteria enabling drug sensitivity determination in less than 24 hours. Detection requires no substrate addition, fewer than 100 cells can be identified, and resistant bacteria can be detected within mixed populations. Fluorescence withstands fixation by paraformaldehyde providing enhanced biosafety for testing MDR-TB and XDR-TB infections.

## Introduction

Tuberculosis (TB) is a major cause of human mortality with 9 million new cases and nearly two million deaths annually; approximately two billion peoples are infected with the causative agent, *Mycobacterium tuberculosis* [1]. While *M. tuberculosis* infections can be effectively resolved with a standard 6–9 month course of antibiotics with at least three drugs, the emergence of drug resistant strains severely complicates treatment. Of particular concern are those strains that are resistant to two or more of the first-line anti-tuberculosis drugs, including multidrug resistant (MDR) – resistant to rifampicin and isoniazid – and extensively drug resistant-tuberculosis (XDR) strains that are resistant to rifampicin, isoniazid, a second line injectable drug (capreomycin, kanamycin or amikacin) and a fluoroquinolone [2], [3]. The devastating impact of XDR TB infections – especially in combination with HIV infection – was demonstrated by an outbreak in Tugela Ferry, KwaZulu-Natal, South Africa, in which 52 of 53 patients died with a median time-to-death after sputum collection of 16 days [4].

A variety of diagnostic tools have been developed for detection and drug susceptibility testing of *M. tuberculosis*. The cornerstone is sputum smear microscopy, a cheap and simple test to detect the presence of acid-fast bacilli in patient's sputum, but which requires a minimum of 10,000 bacteria per ml of sputum, and cannot simply discern drug susceptibilities [1], [5]. In contrast, drug resistance profiles can be readily determined using sensitive and automated radiometric methods or by DNA-based technologies, but these can be time-consuming and expensive, limiting their applicability in the developing world where the vast majority of TB cases occur [5]. There is therefore a need for new diagnostic approaches that combine speed (time-to-detection), sensitivity, specificity, biosafety, and rapid and accurate determination of resistance to the commonly used anti-tuberculosis drugs. Development of such methods is complicated by the extreme slow growth of *M. tuberculosis* (doubling time 24 hrs), the presence of non-tuberculosis strains contaminating patient samples, the dangers of handling highly infectious agents, and the broad-range of bacterial densities in sputum samples. None of the currently available approaches behave perfectly in all facets of an ‘ideal’ diagnostic test.

Mycobacteriophages – viruses of mycobacterial hosts – offer several potential advantages to tuberculosis diagnostic strategies, in that they efficiently and specifically infect and replicate in mycobacteria, and several phage-based approaches have been developed. The commercially available phage amplification biological assay (FASTPlaque™, Biotech Labs Ltd) utilizes *M. tuberculosis*-dependent reproduction of phage D29 and determination of viral particle counts on the fast-growing *Mycobacterium smegmatis* [6], [7], and has been adapted for determining resistance to rifampicin [8]. The luciferase reporter phage assay uses recombinant mycobacteriophages carrying the firefly luciferase gene to detect *M. tuberculosis* by luminescence, coupled with empirical determination of drug resistance by light emission in the presence of antibiotic [9]. Both methods are rapid, accurate, and simple, but are not well-suited for the detection of partially-resistant cultures, require the propagation of potentially infectious live cultures, and the high level of sensitivity in laboratory grown cultures has yet to be fully replicated with clinical specimens [10]–[12]. A detailed comparison of bacteriophage-based and other tests for detection of *M. tuberculosis* in clinical samples has been described previously [13], [14].

Here we report the construction of a new group of reporter mycobacteriophages that contain the fluorescent reporter genes, *gfp* or *ZsYellow*. These fluoromycobacteriophages have advantageous features in pre-clinical analyses including rapid and sensitive identification of *M. tuberculosis* cells, detection by either fluorescent microscopy or flow cytometry, simple determination of antibiotic susceptibility profiles, and detection of drug resistant cells within mixed cultures. Moreover, fluorescence is maintained for at least two weeks following fixation of samples with paraformaldehyde, increasing biosafety and facilitating storage or transportation of samples during diagnosis.

## Results

### Construction of Fluoromycobacteriophages

Although luciferase reporter mycobacteriophages have been derived from phages TM4, D29, L5, and Che12 [9], [15]–[17], we chose to construct and evaluate fluorophage derivatives of TM4 [18], [19], since shuttle phasmid derivatives of TM4 are relatively simple to manipulate [20], [21], conditionally-replicating mutants of TM4 are available [21], and TM4 luciferase reporter phages have been most extensively evaluated [11], [22]–[25]. TM4 has a broad host-range among mycobacterial strains and infects both fast-growing and slow growing strains including *M. tuberculosis*. TM4 shuttle phasmids – phage-cosmid chimeras – have been used to deliver transposons [26], allelic exchange substrates [27] and reporter genes, and its complete genome sequence is available [19]. The conditionally-replicating derivative phAE87 is unable to form plaques or lyse its hosts at 37°C due to mutations that presumably interfere with DNA replication, but can be propagated at 30°C [21], [26].

**Figure 1:**
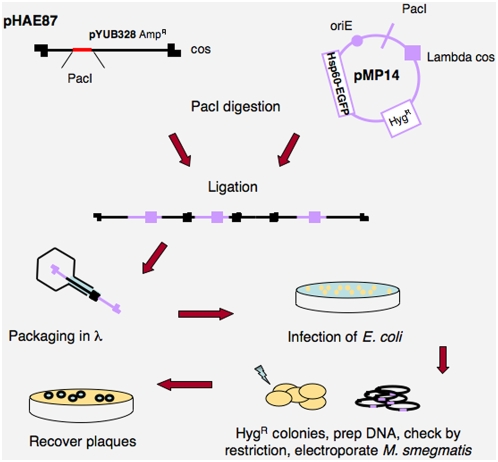
Fluoromycobacteriophages construction. Schematic representation of phAE87::*hsp60-EGFP* construction. Shuttle phasmid phAE87 is a conditionally replicating derivative of phage TM4 in which the cosmid moiety is flanked by Pac I restriction sites. A plasmid derivative of pYUB854 containing the EGFP gene (pMP14) was used to replace the cosmid in phAE87 followed by lambda packaging and recovery in *E. coli*.

To construct fluorophage derivatives of TM4, *gfp* or *ZsYellow* genes were fused to the constitutive *M. bovis* BCG *Hsp60* promoter in plasmid derivatives of pYUB854 [27] and transferred into the phAE87 shuttle phasmid to generate phAE87::*hsp60-EGFP* and phAE87::*hsp60-ZsYellow* respectively (Figure 1). DNA was extracted from the *E. coli*-propagated phasmids and their structures verified by restriction analysis. High titer stocks (>10^10^pfu/ml) were prepared from individual plaques following transfection of *M. smegmatis*, and used in subsequent assays.

### Fluorescent detection of *M. smegmatis* using fluoromycobacteriophages

**Figure 2:**
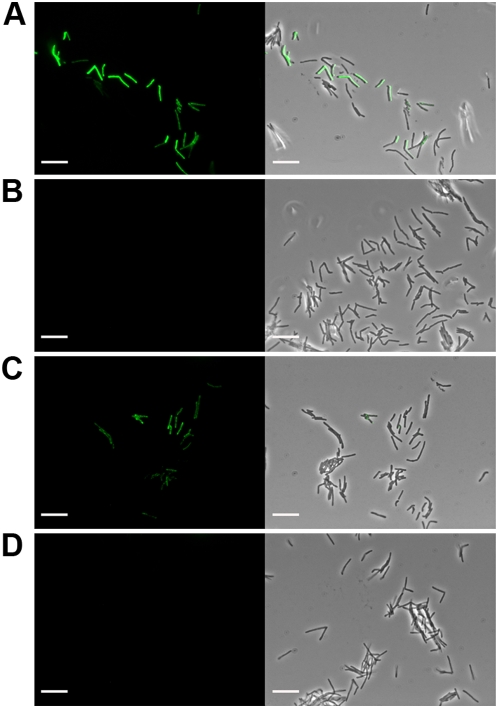
Detection of *M. smegmatis* mc^2^155 cells with fluoromycobacteriophages. Infection of *M. smegmatis* with A, phAE87::*hsp60-EGFP*; C, phAE87::*hsp60-ZsYellow*, or B and D, mock infected controls. Left, fluorescence micrograph images; right, merged fluorescence and phase contrast images. Scale bar, 10 µm.

**Figure 3:**
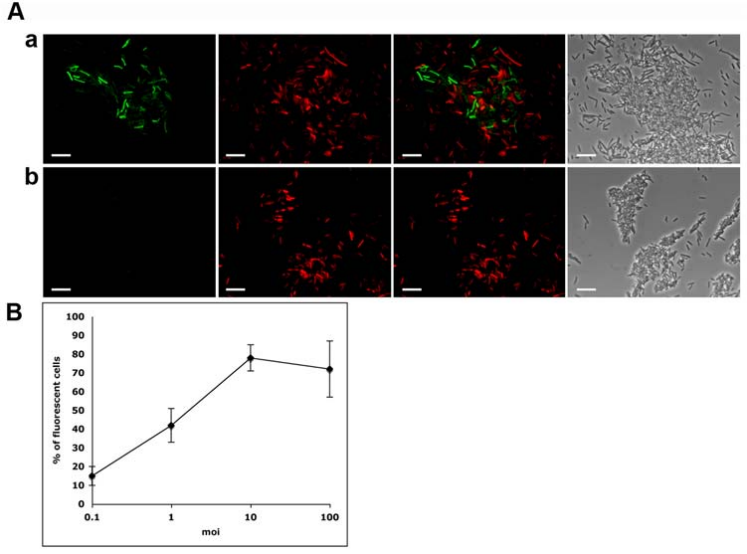
Specificity and efficiency of fluoromycobacteriophage infections. A. A mixture of *E. coli* cells expressing DsRed-Express and *M. smegmatis* were infected with (a) phAE87::*hsp60-EGFP* or (b) mock infected. From left to right: fluorescence micrograph images using a ZsGreen1 filter (green); fluorescence micrograph images using a HQ:R NX filter (red), merged fluorescence images; phase contrast images. Red cells: *E. coli*; green cells: *M. smegmatis* mc^2^155 infected with pHAE87::*hsp60-EGFP*. Scale bar, 10 µm. B. *M. smegmatis* mc^2^155 cells were infected with phAE87::*hsp60-EGFP* at different multiplicities of infection (moi). Green cells were counted in 15 individual fields and the percentage of fluorescent cells compared to the total number of cells in phase contrast images determined.

To evaluate the ability of phAE87::*hsp60-EGFP* and phAE87::*hsp60-ZsYellow* to confer fluorescence following infection, a mid-logarithmic culture of *M. smegmatis* mc^2^155 was infected at a multiplicity of infection (moi) of approximately 100, incubated for 4 hours at 37°C, spun and washed, and examined by fluorescence microscopy (Figure 2). Little or no fluorescence was detected from uninfected cells, and individual cells could be easily detected following infection with either phage (Figure 2). To confirm that fluorescence was specific to infection of *M. smegmatis*, fluoromycobacteriophages were used to infect a mixed culture containing approximately equivalent amounts of *M. smegmatis* and an *Escherichia coli* strain expressing the fluorescent reporter gene *DsRed* (Figure 3). Green fluorescence was readily observed only in infected *M. smegmatis* cells and in each case was distinct and non-overlapping with the red fluorescence from *E. coli* (Figure 3A panels a and b,).

In the experiments shown in Figure 2, approximately 70% of the cells fluoresce following infection with fluoromycobacteriophages. To determine the influence of the multiplicity of infection (moi) on the proportion of fluorescent cells, *M. smegmatis* was infected at a range of moi's from 0.1 to 100 (Figure 3B). Optimal efficiencies of fluorescence were observed at an moi of approximately 10, with fewer fluorescent cells at lower moi's and equivalent proportions at moi's above 10 (Figure 3B). At no moi were more than approximately 75% of the cells infected, and it is not clear if the other 25% of cells failed to fluoresce because they are dead, dormant, uninfectable by TM4, or are infected but do not express the reporter gene; similar results were obtained for both phAE87::*hsp60-EGFP* and phAE87::*hsp60-ZsYellow*. It should be noted that these *M. smegmatis* cultures were grown in the absence of Tween – a detergent commonly used to generate dispersed mycobacterial cultures – because Tween is an inhibitor of TM4 infection. Although cell clumping is therefore one possible cause for the presence of uninfected cells, microscopy shows that a high proportion of bacteria are dispersed as single cells (Figure 2).

**Figure 4:**
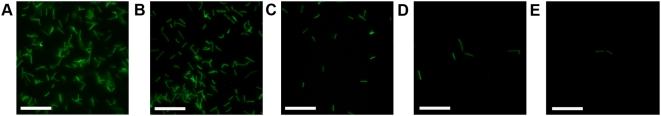
Sensitivity of Fluoromycobacteriophages. Varying numbers of *M. smegmatis* mc^2^155 cells were infected with pHAE87::hsp60-EGFP at a moi of 100. After infection at 37°C for 4 hrs, cells were concentrated by filtration and visualize using by fluorescence microscopy. A, 10^6^; B,10^5^; C, 10^4^; D, 10^3^ and E, 10^2^ cells. Scale bar, 20 µm.

Since a high proportion of cells fluoresce following infection, we reasoned that we should be able to detect relatively small numbers of *M. smegmatis* bacteria. To evaluate this we made dilutions of an *M. smegmatis* culture and infected 200 µl aliquots each with phAE87::*hsp60-EGFP* at an moi of 100. In order to recover all of the bacteria present in the sample, infected cells were collected on a 0.45 µm filter (GH polypro, PALL Life Sciences) and examined directly by fluorescence microscopy (Figure 4). When as few as 100 cells were present in the sample, we could readily recover and identify them by examination of just a few microscopic fields (at 400-fold magnification) (Figure 4E). This experiment shows that infection is sufficiently efficient even when cells are dilute, and that because microscopic detection enables identification of individual infected cells the overall sensitivity of the assay is restricted primarily by the efficiency with which cells can be recovered for microscopy. We note that the opacity of these filters eliminates the ability to determine total bacterial counts using bright field optics.

### Detection of fluorescent cells after paraformaldehyde fixation

**Figure 5:**
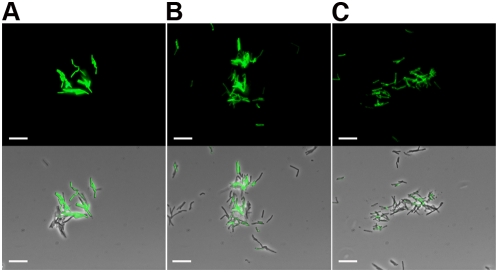
Paraformaldhyde fixation of fluoromycobacteriophage-infected mycobacteria. *M. smegmatis* mc^2^155 cells were infected with phAE87::*hsp60-EGFP* and fluorescence detected in A, live cells, B, fixed cells and C, fixed cells after 2 weeks at 4°C. Top, fluorescence micrograph images; bottom, merge of fluorescence and phase contrast images. Scale bar, 10 µm.

A potential advantage of using GFP as a reporter is that it is known to withstand fixation when expressed in eukaryotic cells [28]. Paraformaldehyde fixation of fluoromycobacteriophage-infected mycobacteria has two key potential advantages. First, it should confer significant biosafety advantages especially when analyzing notably hazardous strains such as XDR-TB, simplifying sample handling procedures such as microscopy and flow cytometry. Secondly, by preserving fluorescence for extended periods of time, samples could be readily stored or transported for off-site analysis. We evaluated this using *M. smegmatis*, adding 2% paraformaldehyde – which effectively kills mycobacteria [29] – after phage incubation. Samples were stored at 4°C for varying periods of time and examined by microscopy. As shown in Figure 5, fluorescence was maintained following fixation and showed only modest decay during a two-week incubation. Incubation for a shorter period (48 hours) at room temperature showed no reduction of fluorescence (data not shown).

### Detection of *Mycobacterium tuberculosis* using fluoromycobacteriophages

**Figure 6:**
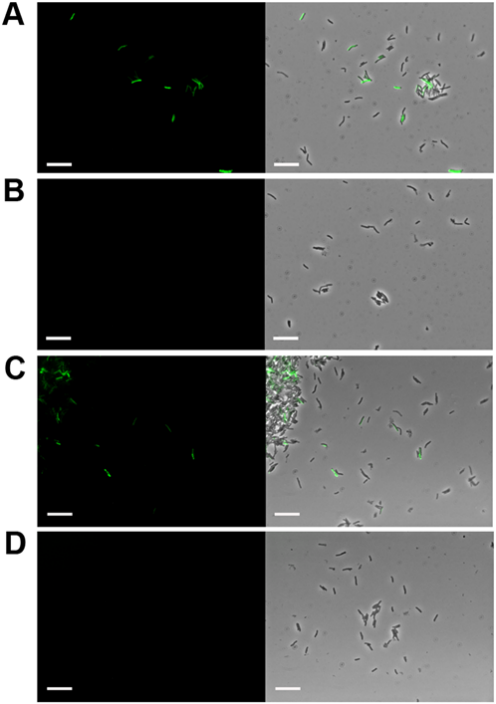
Infection of *M. tuberculosis* mc^2^6230 cells with fluoromycobacteriophages. *M. tuberculosis* was infected with phages as follows: A, phAE87::*hsp60-EGFP*; C, phAE87::*hsp60-ZsYellow*; B and D, mock infected controls. Cells were fixed with 2% paraformaldehyde, washed, and resuspended in PBS prior to microscopy. Left panels, fluorescence micrograph images; right panels, merged fluorescence and phase contrast images. Scale bar, 10 µm.

Using the avirulent strain *M. tuberculosis* mc^2^6230 [30], [31], bacteria could be readily detected by fluorescence following fluoromycobacteriophage infection in a similar assay configuration to that used for *M. smegmatis* (Figure 6). However, in one prominent departure, fluorescence was weak after only 4 hours incubation following infection, and improved greatly after 16 hours (Figure 6); incubation for up to 30 hours provided no further increase in fluorescence. In a standard assay, *M. tuberculosis* cells were incubated overnight with phAE87::*hsp60-EGFP*, fixed with 2% paraformaldehyde, washed, resuspended in buffer, and examined by microscopy. Using a moi of 100, approximately 50% of cells fluoresced, although this proportion varied significantly between experiments and ranged from 37–67% with the highest proportions obtained when cells were grown to late logarithmic growth and in the absence of detergent (Table S1).

### Antibiotic Susceptibility Testing of *M. tuberculosis* with Fluoromycobacteriophages

**Figure 7:**
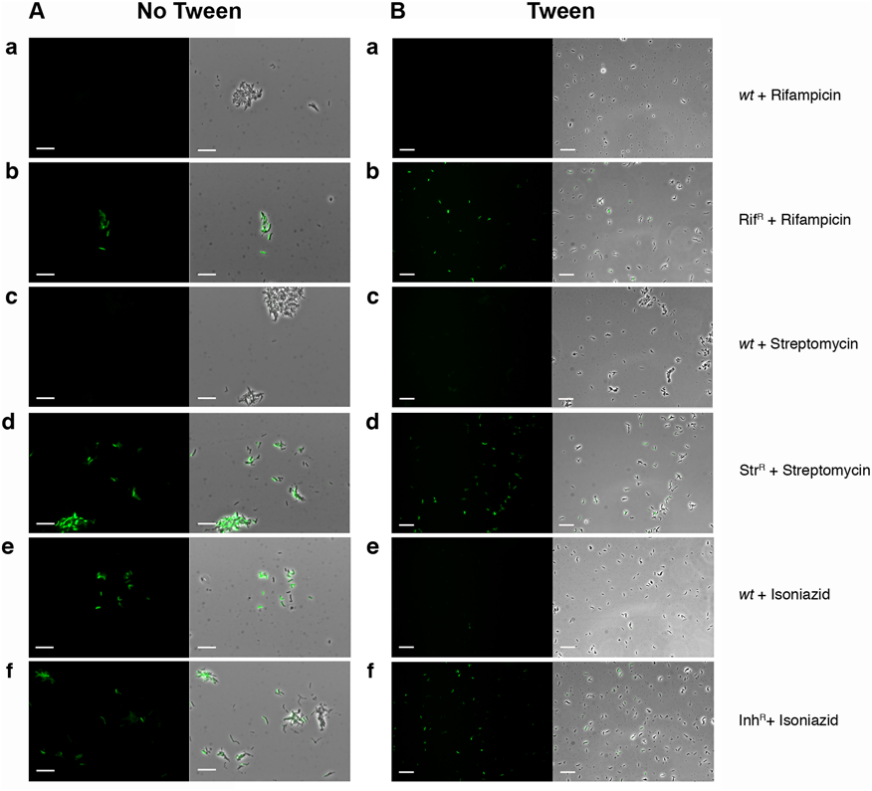
Antibiotic susceptibility testing of *M. tuberculosis* using fluoromycobacteriophages. *M. tuberculosis* mc^2^6230 *wt* and antibiotic resistant strains were infected with phAE87::*hsp60-EGFP* with or without antibiotic addition as indicated. A. Cultures were grown in the absence of Tween, washed, and tested. In panels a–d antibiotics and phage were added simultaneously, incubated for 16 hours, and examined by microscopy. In panels e–f INH was added first for 48 hours followed by phage addition, incubation for a further 16 hours, and examined by microscopy. Scale bar, 10 µm. B. Cultures were grown in the presence of Tween, centrifuged and washed twice, and then incubated with antibiotic (as indicated) for 24 hours. Phage were then added, cultures incubated for 16 hour, and examined by microscopy. Scale bar, 20 µm.

To confirm that *gfp* expression following fluoromycobacteriophage infection is responsive to antibiotics, an *M. tuberculosis* mc^2^6230 culture was grown (in the absence of Tween), spun and washed, and phage added with either rifampicin or streptomycin simultaneously (Figure 7A panels a and c), incubated for 16 hours and examined by microscopy; in both experiments fluorescence was eliminated by antibiotic addition. Isogenic rifampicin-resistant and streptomycin-resistant derivatives of *M. tuberculosis* mc^2^6230 were constructed by recombineering with ssDNA substrates [32] – introducing a S456L substitution in *rpoB* (Rv0667) and a K43R substitution in *rpsL* (Rv0682), respectively – and tested in a similar assay (Figure 7A panels b and d). Antibiotic resistant strains fluoresced well in the presence of antibiotic (Figure 7A panels b and d) demonstrating that fluoromycobacteriophages can provide a report of resistance/susceptible profiles of *M. tuberculosis* to rifampicin and streptomycin in approximately 16 hours. Because isoniazid (INH) is an inhibitor of mycobacterial cell wall synthesis rather then gene expression, cultures were pre-incubated with INH for 48 hours prior to addition of fluoromycobacteriophage; however, fluorescence was unaffected even in INH-sensitive bacteria (Figure 7A, panel e). Although a variety of pre-incubation times and drug concentrations have been examined using cells grown in these conditions, no significant influence of INH on fluorescence was observed (data not shown).

Other investigations have tested INH susceptibilities using cultures grown in the presence of Tween detergent to facilitate cell dispersion [9], so we also tested Tween-grown *M. tuberculosis* cultures (Figure 7B). In these experiments, following growth with Tween, cells were centrifuged, washed twice, and resuspended in the presence of antibiotic. After incubation for 24 hours, phage were added, incubated for a further 16 hours, and examined by microscopy (Figure 7B). Under these conditions, isoniazid as well as rifampicin and streptomycin strongly reduced fluorescence in drug sensitive cells (Figure 7B panels a, c, e). Resistant strains all failed to respond to the addition of antibiotic (Figure 7B panels b, d, f) showing that under these conditions all fluoromycobacteriophages can be used to determine INH as well as rifampicin and streptomycin susceptibility profiles. It is not clear why growth in the presence of Tween would strongly enhance the effect of INH in this assay, although we favor the explanation that it results in inhibition of phage infection, rather than a metabolic function necessary for GFP expression and fluorescence.

### Determination of drug susceptibilities using fluoromycobacteriophages and flow cytometry

**Figure 8:**
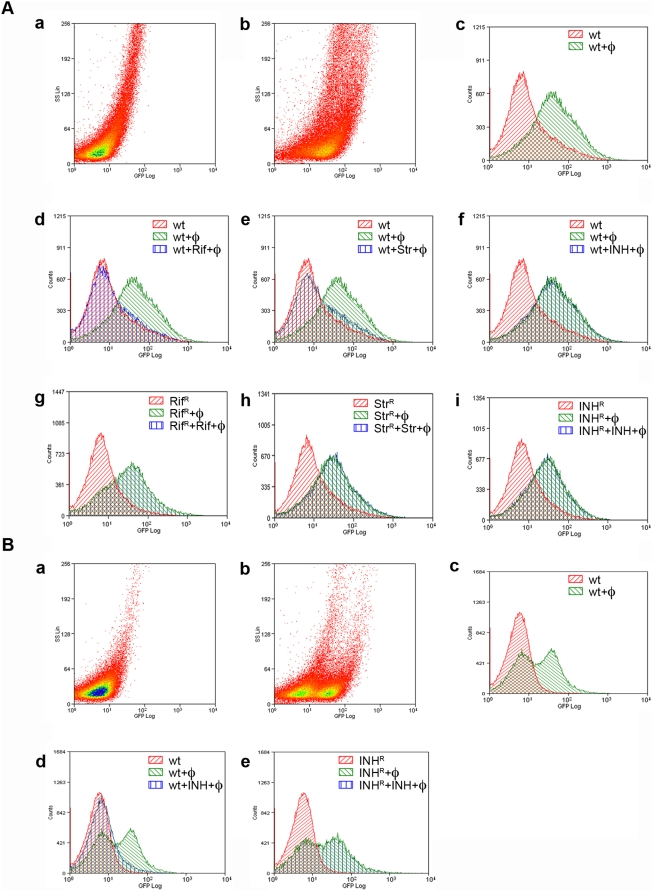
Fluoromycobacteriophage *M. tuberculosis* susceptibility testing using flow cytometry. A. *M. tuberculosis* mc^2^6230 strains grown in the absence of Tween detergent were infected with phAE87::*hsp60-EGFP* with or without antibiotic treatment, fixed with paraformaldehyde, and analyzed by flow cytometry. In panels a, mock-infected control cells and b, phage infected cells, the intensity of fluorescence is plotted against light side scatter. The signal observed in mock-infected cells corresponds to autofluorescence of *M. tuberculosis* and the shift towards greater fluorescence in phage-infected cells shows that a high proportion of cells are infected and expressed GFP. In panels c–i the level of fluorescence is plotted against the number of events counted. In each experiment mock-infected cells are shown in red, phage infected cells in green, and antibiotic-treated phage infected cells in blue. When treated with rifampicin (Rif) or streptomycin (Str) antibiotics were added simultaneously with phage (φ), incubated for 16 hours, fixed and analyzed; for INH treatment cells were pre-incubated with antibiotic for 48 hours, incubated with phage for 16 hours, fixed, and analyzed. B. *M. tuberculosis* mc^2^6230 strains were grown in the presence of Tween, washed, preincubated with isoniazid for 24 hours, infected with phAE87::*hsp60-EGFP*, fixed, and analyzed by flow cytometry. Panels a–e correspond to panels a–c, f, and i of Figure 8A respectively.

Flow cytometry offers an alternative approach to the detection of fluorescence following fluoromycobacteriophage infection. While the instrumentation required might seem prohibitively costly for routine use in many areas of the world where the incidence of tuberculosis is high, if the HIV infection rates are also high, flow cytometry may have been previously established for CD4 count determination; the province of KwaZulu-Natal in South Africa is one example of this. To evaluate this approach, *M. tuberculosis* cells infected with phAE87::*hsp60-EGFP* cells were fixed, and analyzed by flow cytometry to measure side light scatter as well as fluorescence (Figure 8A). Although *M. tuberculosis* has significant autofluorescence as seen in the mock-infected cells (Figure 8A panels a and c), fluoromycobacteriophage-infected cells can be readily distinguished (Figure 8A panels b and c) and a very high proportion of cells fluoresced following fluoromycobacteriophage infection (Figure 8).

Addition of either rifampicin or streptomycin simultaneously with phage to a drug sensitive strain shifted the signal observed with phage-infected cells to one superimposable with uninfected cells (Figure 8A panels d and e), whereas addition to antibiotic to a resistant strain did not alter the profile of expression from untreated cells (Figure 8A panels g and h). The drug susceptibility profiles were therefore clearly discerned and the fluorescent patterns strongly reflect those observed by microscopy (Figure 7). When similar assays were performed with as few as a total of 10^4^ bacteria, the efficiency of phage infection was reduced somewhat although susceptibilities to rifampicin and streptomycin could be easily determined (Figure S1). When the influence of INH addition was examined a similar response to that shown in Figure 7 was seen. When cells were grown in the absence of tween, no INH inhibition of fluorescence (Figure 8A panel f) was observed. However, when grown with tween, INH strongly inhibited fluorescence, although the proportion of infected cells was also affected (Figure 8B). We also observed that fluorescence is responsive to addition of kanamycin generating profiles indistinguishable to rifampicin- or streptomycin-treated sensitive cells (Figure S2). While ethambutol, ethionamide and ofloxacin also inhibited fluorescence, strong antibiotic inhibition was dependent on growth in tween, and complete inhibition by ofloxacin required more extensive pre-incubation in the presence of antibiotic (Figure S2).

### Analysis of partially resistant *M. tuberculosis* cultures

**Figure 9:**
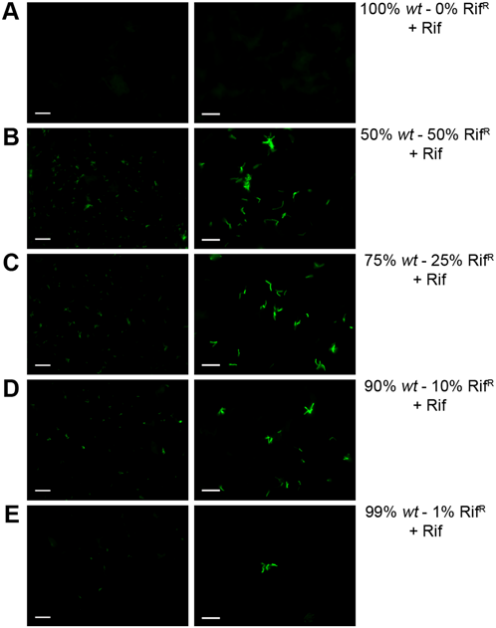
Infection of a mixed culture of antibiotic resistant and sensitive strains. *M. tuberculosis* mc^2^6230 *wt* was mixed with the isogenic Rif^R^ strain at different proportions. A total of 10*^6^* mixed cells were infected with phAE87::*hsp60-EGFP* in the presence of Rifampicin; cells were fixed, collected by filtration and analyzed by fluorescent microscopy. (A) 100% *wt*: 0% Rif^R^ (B) 50% *wt*: 50% Rif^R^; (C) 75% *wt* :25% Rif^R^ ; (D) 90% *wt* :10% Rif^R^; (E) 99% *wt* :1% Rif^R^. Left panels, fluorescence micrograph images; scale bar 20 µm; right panels, fluorescence micrograph images, scale bar 10 µm.

**Figure 10:**
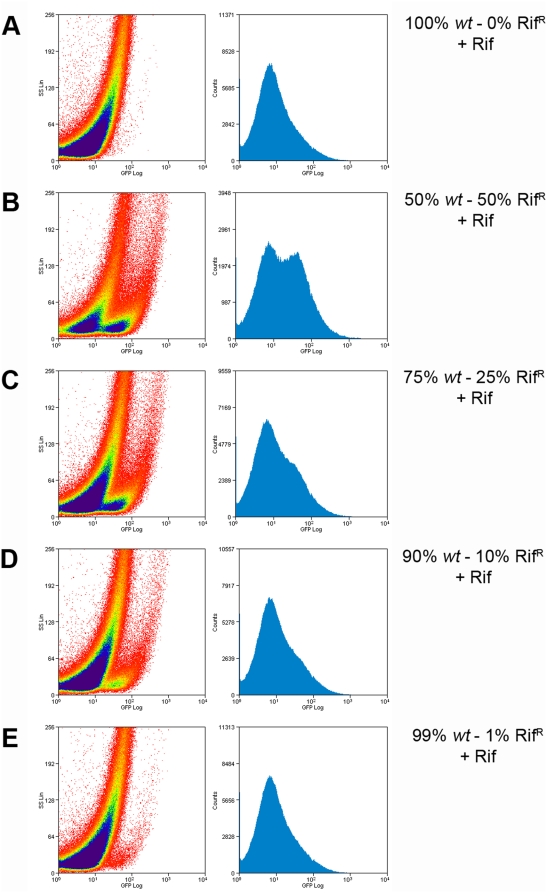
Detection of *M. tuberculosis* mc^2^6230 Rif^R^ cells in a mixed population using flow cytometry. *M. tuberculosis* mc^2^6230 *wt* was mixed with the isogenic rif^R^ strain at different proportions. A total of 10*^7^* mixed cells were infected with phAE87::*hsp60-EGFP* in the presence of Rifampicin; cells were fixed and analyze by flow cytometry. (A) 100% *wt*: 0% Rif^R^ (B) 50% *wt*: 50% Rif^R^; (C) 75% *wt* : 25% Rif^R^ ; (D) 90% *wt* :10% Rif^R^; (E) 99% *wt* :1% Rif^R^ . Left panels, the intensity of fluorescence is plotted against light side scatter; right panels, the level of fluorescence is plotted against the number of events counted.

Because sputum samples may not be phenotypically homogenous cultures, we evaluated the ability of fluoromycobacteriophages to detect drug resistant strains within mixed cultures. Rifampicin-resistant and rifampicin-sensitive cells were mixed at varying ratios, and tested with phAE87::*hsp60-EGFP* in the presence of rifampicin (Figure 9). To optimize recovery of bacteria present in the sample, cells were fixed and recovered on a 0.45 µM filter after phage infection as described above. In samples containing a total of approximately 10^6^ cells in a 200 µl aliquot, rifampicin-resistant cells could be readily detected when they constituted as few at 1% of the total population using a 400× or 1000× fold magnification (Figure 9E). A similar experiment was performed using flow cytometry and similar results were observed with detection of a very small population (1%) of rifampicin resistant cells when a mixed culture of sensitive and resistant cells was infected in the presence of rifampicin (Figure 10). We note that the number of drug-resistant cells rather than the total number of bacteria determines the sensitivity of detection in such mixed cultures.

### Direct drug susceptibility testing of *M. tuberculosis* colonies

The ability to rapidly determine the drug susceptibility profiles of *M. tuberculosis* colonies grown on solid medium is potentially useful in clinical settings as well as in the research laboratory. To test this, single *M. tuberculosis* colonies were resuspended in a small volume (400 µl) of media and the suspension passed several times through a syringe to disperse clumps. Phage and antibiotics were added simultaneously, incubated for 16 hours, fixed, collected on a filter, and analyzed by fluorescence microscopy. Strong fluorescence was observed with accurate reporting of the drug susceptibility profiles for rifampicin, streptomycin and kanamycin (Figure S3).

## Discussion

The emergence of MDR and XDR TB strains and the problems encountered in controlling these infections emphasizes the need for a rapid, sensitive, and accurate diagnostic test for tuberculosis drug susceptibility testing. Here we have shown that fluoromycobacteriophages have features suggesting they have potential advantages over current diagnostic tools including luciferase reporter phages and phage amplification assays. Fluoromycobacteriophages are sensitive to commonly used anti-tuberculosis drugs including rifampicin, streptomycin and kanamycin – with the notable exception of INH when grown in the absence of Tween – and readily discriminate between drug resistant and sensitive strains. The assay does not require addition of any exogenously added substrates and chemical fixation provides a high level of biosafety, reducing major risks in analyzing potentially infectious agents by both microscopy and flow cytometry. The rapid time-to-death reported for XDR-TB patients in Tugela Ferry [4] – coupled with capable transmission of these strains [33] – emphasizes the need for a rapid drug susceptibility test that can be performed locally, rather than in centralized facilities. While DNA-based assays provide a rapid and effective approach it is less clear if these can be performed at the point of patient contact with drug susceptibility determination within a sufficient time frame necessary to treat TB infections with an appropriate drug regimen, and that avoids XDR-TB transmission.

The pre-clinical properties of the fluoromycobacteriophages satisfy the criteria for a simple, rapid, sensitive, safe, and accurate test for tuberculosis drug susceptibilities. Evaluation of this technology in a clinical setting will require optimization of sputum processing for efficient phage infection, and we note that while sputum processing methods have been described for use of luciferase reporter phages and commercially available FastPlaque™ phage amplification assays [11], [34], efficient infection of mycobacteria derived from sputum has thus far limited replication of the pre-clinical performance of these methods in clinical settings [13], [14]. Efficient recovery of phage-infectable *M. tuberculosis* cells from sputum is thus anticipated to be a key factor in clinical implementation of the fluoromycobacteriophage assay described here, as it is for other phage-based assays. An additional limitation of using phage TM4 as a fluoromycobacteriophage platform is that it does not discriminate between *M. tuberculosis* and non-tuberculous mycobacterial strains. This could potentially be addressed by either switching to an alternative phage platform with a more restricted host range, by engineering of TM4, or through addition of hydroxypropiophenone (NAP) that selectively inactivate *M. tuberculosis* complex bacteria [11]. However, we also note that TM4 has a potential advantage in that it efficiently infects mycobacterial cells that have entered stationary phase as a function of a peptidoglycan hydrolyzing motif in the phage tapemeasure protein [35].

Fluorescence microscopy coupled with compatible filters – which are also available in a multi-well format – enables the detection of small numbers of cells using a low power objective lens and larger microscope fields [36], [37]. Although fluorescent microscopy may be prohibitively costly in many areas of high tuberculosis incidence due to its cost and short life spans of light sources, two facets of the fluoromycobacteriophage assay suggest potential solutions. First, because samples can be fixed prior to slide preparation without loss of fluorescence, microscopy could be performed at a central facility serving nearby communities, even though this would extend the total time to obtain assay results. Secondly, less expensive and simpler fluorescent microscopes using light emitting diodes (LED) have been developed for the use of auramine-stained *M. tuberculosis* samples [38], and these could readily be adapted for detection of fluoromycobacteriophage infected cells.

Flow cytometry has been described previously for *M. tuberculosis* diagnosis [39] and has the advantage that flow cytometers are already in use in many areas of high HIV incidence. For example, *M. tuberculosis* can be detected by fluorescence from hydrolysis of fluorescein diacetate, and susceptibilities to isoniazid, ethionamide and rifampicin determined in 24 hours, although aerolisolization of unfixed viable cells is a significant concern [40]. Safe cytometric methods using fluorescent dyes to measure bacterial growth in the presence or absence of antibiotics have also been described although they require somewhat longer times-to-detection (72 hours) [41], [42]. Mycobacteriophages have some advantage over these methods by virtue of their specificities for mycobacterial hosts such that fluorescence is dependent on phage infection. Furthermore, flow cytometry provides simple discrimination of drug resistant and drug sensitive *M. tuberculosis* cells, with high sensitivity of detection. Using the pre-clinical assay configuration in which rifampicin, streptomycin or kanamycin are added simultaneously with phage, a large number of samples can be analyzed in a short period of time (<1 minute for each sample by flow cytometry), and drug susceptibilities determined within 16 hours. Importantly, fixation of the sample prior to analysis overcomes substantial biosafety concerns experienced with other methods for drug susceptibility determination using flow cytometry [40]. Finally, flow cytometric analysis of fluoromycobacteriophage infected cells is not significantly impeded by *M. tuberculosis* clumping, even when grown in the absence of Tween detergent.

Responsiveness of the fluoromycobacteriophage assay to rifampicin and streptomycin was not unexpected, since these antibiotics are known to inhibit mycobacterial gene expression; fluorescence is also strong inhibited by kanamycin (Figure S2), as well as by ciprofloxacin and sparfloxacin (Liliana Rondon Salazar and Howard Takiff, personal communication). The responsiveness to INH is more complex, and when cells are grown in the absence of Tween, INH addition does not significantly inhibit fluorescence in any configuration that we have tested. In contrast, growth in Tween – followed by INH preincubation for 24 hrs and phage addition leads to strong abrogation of GFP fluorescence in sensitive, but not resistant cells. This suggests that fluoromycobacteriophages can be used to evaluate INH susceptibilities although the requirement for pre-incubation with Tween may extend this assay to longer than 48 hours. This assay was further complicated by the presence of a sub-population of cells (0.1–1%) that fluoresced in the presence of INH and could be detected microscopically; these likely correspond to persisters that display INH tolerance due to their metabolic state (C. Vilcheze and WRJ; unpublished observations). Finally, we note that fluorescence was also not subject to INH inhibition when individual colonies from solid media were tested, although these were also analyzed in the absence of Tween growth and a significant number of cells may not be in a growth-dependent INH-sensitive state. The use of INH-responsive promoters [43] may be useful for addressing this.

In summary, fluoromycobacteriophages offer a potentially powerful new approach to the detection and antibiotic susceptibility testing of tuberculosis infections, combining established methods for phage-based testing with microscopic or flow cytometry approaches. The ability to directly visualize the drug susceptibility profile allows for the analysis of small numbers of cells, is both specific and fast, and can detect resistant strains within mixed populations; rapid detection of rifampicin resistance as a marker for suspected MDR-TB and XDR-TB [44] would be especially useful. We reported the detection of just 1% of resistant cells to rifampicin in a mixed population, comparable to the sensitivity of the agar proportion method on Lowenstein-Jensen medium and Middlebrook 7H11 agar that is an established standard for susceptibility testing of *M. tuberculosis* [45].

## Materials and Methods

### Bacterial strains

*M. smegmatis* mc^2^155 has been described previously [46]. *M. tuberculosis* mc^2^6230 is a derivative of H37Rv and contains a deletion of the RD1 region and *panCD*, resulting in a pan^−^ phenotype [31]. All culture media were supplemented with panthothenate (100 µg ml^−1^) and the following antibiotics when required: kanamycin (20 µg ml^−1^ or 4 µg ml^−1^ for AST), streptomycin (6 µg ml^−1^), rifampicin (2 µg ml^−1^), isoniazid (0.4 µg ml^−1^), ethionamide (10 µg ml^−1^), ethambutol (5 µg ml^−1^) and ofloxacin (10 µg ml^−1^). For AST, drug concentrations were optimized for discrimination between resistant and susceptible strains using fluoromycobacteriophages.

### Construction of antibiotic resistant mutants

Isogenic rifampicin and streptomycin resistant mutants of *M. tuberculosis* mc^2^6230 were constructed by ssDNA recombineering [32]. Plasmids pJV114F or pLAM12 were introduced into *M. tuberculosis* mc^2^6230 by electroporation, and strains were grown and induced for recombineering functions as described previously [32]. Plasmid pJV114F is a derivative of pJV62 [32] containing a counterselectable *sacB* gene. The following ssDNA oligonucleotides were used for electroporation: JCV327 5′ CCCGCTGTCGGGGTTGACCCACAAGCGCCGACTGTtGGCGCTGGGGCCCGGCGGTCTGTCACGTGAGCGTG 3′ and JCV328 5′ CACGCTCACGTGACAGACCGCCGGGCCCCAGCGCCaACAGTCGGCGCTTGTGGGTCAACCCCGACAGCGGG 3′ to target *rpoB* (Rv0667) and generate a S456L mutation; and JCV329 5′ TGGTGTATGCACCCGCGTGTACACCACCACTCCGAgGAAGCCGAACTCGGCGCTTCGGAAGGTTGCCCGCG 3′ and JCV330 5′ CGCGGGCAACCTTCCGAAGCGCCGAGTTCGGCTTCcTCGGAGTGGTGGTGTACACGCGGGTGCATACACCA 3′ to target *rpsL* (Rv0682) and generate a K43R mutation. Electroporations were performed with 100 ng of each ssDNA oligonucleotide and recovered for 3 days in 7H9 medium supplemented with Middlebrook OADC Enrichment (Becton Dickinson and Company Sparks, MD), Tween-80, panthothenate (100 µg ml^−1^) and plated on 7H10 agar containing OADC, panthothenate (100 µg ml^−1^) and the appropriate antibiotic. To cure the strains of plasmid pJV114F, isolated colonies were grown in liquid media in the absence of kanamycin until saturation and sub-cultured at high dilution. After growth, cells were plated on 7H10 OADC, panthothenate (100 µg ml^−1^) with the appropriate antibiotic plates in the presence of 10% sucrose; loss of pJV114F was corroborated by patching single colonies to sucrose plates in the absence and presence of kanamycin. In each mutant the *rpoB* and *rpsL* region was sequenced to confirm the presence of the desired point mutation. A similar approach was used to generate an isoniazid mutant, although the high level of spontaneous resistant mutants was problematic. We therefore characterized a spontaneous *M. tuberculosis* mc^2^6230 INH^R^ mutant and after sequencing possible known gene targets we identified a point mutation in *katG* (Rv1908c) (H104Q).

### Construction of fluoromycobacteriophages

Fluoromycobacteriophage phAE87::*hsp60-EGFP* was constructed as follows: an Xba I DNA fragment from pJL37GFP [47] containing the *EGFP* gene under control of the BCG *Hsp60* promoter was cloned in pYUB854 [27] to generate plasmid pMP14, which was used to replace the pYUB328 vector moiety in shuttle phasmid phAE87. In brief, phAE87 and pMP14 were digested with Pac I, ligated and packaged in lambda heads *in vitro* using the Gigapack III XL-7 (Stratagene), and used to transduce *E. coli* HB101. Hygromycin^R^ clones were selected and phasmid DNAs prepared and characterized by restriction. One phasmid was used to transfect *M. smegmatis* mc^2^155 by electroporation[27], individual plaques were tested to confirm the presence of *EGFP* and a large high titer (10^12^ pfu/ml) phage stock from a purified plaque was prepared.

A similar strategy was used to construct pHAE87::*hsp60-ZsYellow*, by PCR amplification of *ZsYellow* from plasmid pZsYellow (Clontech Laboratories, Inc.) using primers ZsYellowF 5′ GGAATTCCATATGGCTCATTCAAAGCACGGT 3′ and ZsYellowR 5′ CAGTTGGAATTCTAGAGTCGC 3′. The amplicon was digested with Nde I and EcoR I and inserted in pJL37 under the control of BCG *Hsp60* promoter. After restriction with Xba I, a fragment containing *Hsp60-ZsYellow* was cloned in pYUB854 generating plasmid pMP15 and inserted into phAE87 as described above. All phage dilutions were made in phage buffer supplemented with 3 mM CaCl_2_.

### Infection of *M. smegmatis* mc^2^155 with fluoromycobacteriophages

*M. smegmatis* mc^2^155 cells were grown to an OD_600 nm_ of 1 in 7H9+ADC in the absence of Tween. Approximately 200 µl of cells were mixed with 100 µl of a phage dilution to obtain an moi of 100 (or as required), incubated without shaking for 15 min at 37°C and then for 4 h at 37°C with moderate shaking. Five µl were spotted onto a glass microscope slide, covered with a glass coverslip, sealed with melted mix of 1∶1∶1 vaseline∶ lanoline∶ paraffin, and examined by fluorescence microscopy. For cell fixation, after incubation for 4 h at 37°C 1 volume of 4% paraformaldehyde in PBS was added and the mix incubated for 30 min at room temperature. Cells were washed with 10% glycerol twice and resuspended in PBS (∼1/10 of original volume) and examined by microscopy as above.

### Moi and percentage of cells infected, specificity and sensitivity

*M. smegmatis* mc^2^155 cells were infected as described above with the appropriate amount of phage to obtain a multiplicity of infection (moi) of 100, 10 , 1 or 0.1. After infection, 5 µl of the mix were spotted on a slide before examination under the microscope. Fifteen individual fields for each moi used were recorded and the percentage of fluorescent cells compared to total cells in phase contrast images was calculated. The % mean±SD was calculated for each moi.

*E. coli* DH5α competent cells were transformed with pDsRed-Express (Clontech Laboratories, Inc.), grown to exponential phase and mixed with a culture of *M. smegmatis* mc^2^155. The mixed culture was infected with pHAE87::*hsp60-EGFP* for 4 hours at 37°C, cells were fixed as described above and 5 µl examined under a fluorescence microscope using a red or green filter.

To evaluate the sensitivity of detection, 200 µl of 10-fold dilutions of *M. smegmatis* mc^2^155 cells (initial cell number 1×10^8^/ml) were infected with the appropriate amount of pHAE87::*hsp60-EGFP* to maintain a moi of 100 for 4 hours at 37°C. After infection, cells were fixed with paraformaldehyde as described above, filtered through a GH polypro 0.45 µm filter, 13 mm (PALL Life Sciences) and washed three times with one volume of PBS. The damp filter was placed on a microscope slide and immersion oil and a cover slip were placed on top [48]and sealed as described above. Ten fields were examined to determine the minimal number of cells that could be detected.

### Detection and antibiotic susceptibility testing (AST) of *M. tuberculosis* mc^2^6230 with fluoromycobacteriophages

*M. tuberculosis* mc^2^6230 cells were grown until an OD_600 nm_ of 1 in 7H9+OADC+0.05% Tween 80+panthothenate in the presence of carbenicillin (CB) (50 µg ml^−1^) and cycloheximide (CHX) (10 µg ml^−1^). When antibiotic resistant strains were used, the appropriate antibiotic was added to the culture at the concentrations indicated above. Cells were washed twice in fresh media (7H9+OADC without Tween and CB and CHX) and resuspended in the same media at an OD_600 nm_ of 0.5. When performing AST, antibiotics were added at this step. Cells were incubated standing for 24 hours (when RIF and STR was used) and 24–48 hours for INH. This step is necessary to eliminate traces of Tween that inhibit phage infection and was used to pre-incubate the cells with antibiotic. After that period, cells were spun down and resuspended in fresh media in the presence of antibiotic. For infection of cells grown in the absence of Tween the protocol described for flow cytometry detection was followed (described in detail below). Approximately 200 µl of cells were mixed with 100 µl of a phage dilution to obtain a moi of 100. The mix was incubated standing for 15 min at 37°C and for 16–18 h (overnight) at 37°C with moderate shaking. For all experiments using *M. tuberculosis* mc^2^6230 cells were fixed and washed as described for *M. smegmatis* after incubation with paraformaldehyde for 1.5 hours. Counting of colony forming units (CFU) was routinely done after this incubation to ensure that no viable cells were present. For assays using mixed cultures (*wt* and antibiotic resistant strains), cells were mixed at the indicated proportion before preincubation with the antibiotics.

To infect cells from a colony, cultures from *M. tuberculosis* mc^2^6230 *wt* and antibiotic resistant strains were streaked out on 7H11 plates and incubated for 2–3 weeks at 37°C. A single colony was resuspended in 400 µl of 7H9+OADC+panthothenate, vortexed for 2 minutes and passed several times through a syringe with a 23 gauge needle. Cell suspensions were left standing for 5 minutes and 200 µl of the supernatant were mixed with 100 µl of a phage dilution to obtain a moi of 100 (assuming about 10^6^ cells/colony). For AST, phage and antibiotics were added at the same time. The mix was incubated standing for 15 min at 37°C and for 16–18 h (overnight) at 37°C with moderate shaking. After that, cells were fixed with paraformaldehyde 2% for at least 1.5 hrs. Cells were concentrated by filtration as described above and examined by fluorescence microscopy.

### Microscopy and settings

A fluorescence microscope (Axiostar Plus; Carl Zeiss) with a 40× objective and a 100× objective with oil immersion and phase contrast was used. Fluorescent images were acquired using an AxioCam MRc5 camera (Carl Zeiss) and Carl Zeiss AxioVision Rel. 4.6 software. In all experiments the same exposure time was used for infected cells and mock infected cells. For detection of fluorescent proteins the following filters were used: for EGFP, CLON ZsGreen1 (42002- HQ 470/30X, HQ 520/40m, Q495LP); for ZsYellow, CLON ZsY1 (42003- HQ500/35X, HQ550/40m, Q525LP); for DsRed-Express, HQ:R NX (41002c- HQ545/30X, HQ620/60m, Q570LP) from Chroma Technology Corporation. Image processing was performed using Adobe Photoshop CS2 (Adobe Systems Incorporated), but maintaining identical brightness and contrast settings for images of infected cells and controls.

### Flow Cytometry

*M. tuberculosis* mc^2^6230 *wt* and antibiotic resistant strains were grown until saturation in the presence of Tween as described above, sub-cultured in 10 ml of 7H9+OADC panthothenate+CB+CHX without Tween at an initial OD_600 nm_ of 0.1 and grown standing until an OD_600 nm_ of about 1; antibiotics were added at the indicated concentrations as appropriate. Cells were spun down and resuspended in fresh media (without CB and CHX) to an OD_600 nm_ of 0.5 and passed several times through a syringe with a 23 gauge needle. Phage infections were performed as described above for the colony protocol, but after fixation cell suspensions were kept at 4°C until use, no washing step was necessary before injection of the samples in the flow cytometer. Analysis was performed with a Beckman-Coulter CYAN ADP (Beckman-Coulter, Fullerton, CA) flow cytometer with Summit 4.3 software (Dako Colorado Inc.).

To assay the minimum number of cells that could be detected by flow cytometry, serial dilutions were made and 100 µl of cells were infected with a small amount (0.25 µl) of a phage concentrated stock (10^13^ PFU/ml). Infection was done in the presence of 1 mM CaCl_2_ and cells incubated as described above; 100 µl of paraformaldehyde 4% (2% final concentration) was added to fix cells for at least 1.5 hrs before analysis.

## Supporting Information

Table S1 (0.08 MB PDF)

Figure S1 (0.31 MB PDF)

Figure S2 (0.76 MB PDF)

Figure S3 (0.17 MB PDF)
