# Supplementary material for: Fluoromycobacteriophages for Rapid, Specific, and Sensitive Antibiotic Susceptibility Testing of Mycobacterium tuberculosis
Source: PLoS One. 2009 Mar 20;4(3):e4870. doi: 10.1371/journal.pone.0004870 (PMC2654538; doi:10.1371/journal.pone.0004870)
Supplement: Table S1 — (0.08 MB PDF) [file pone.0004870.s001.pdf]

Table S1: **Percentage of *M. tuberculosis* mc^{2}6230 cells infected with pHAE87::*Hsp60-EGFP***

M. tuberculosis mc^{2}6230 cells were infected with pHAE87::*Hsp60-EGFP* after growth until mid or late exponential phase in the presence or absence of Tween. About 10 individual fields for each condition were recorded and the percentage of fluorescent cells compare to total cells was calculated. The % mean ± SD was calculated.

**OD_{600nm}**	**Growth medium**	**% fluorescent cells**
0.6	7H9+OADC	51 ± 9
	7H9+OADC+Tween	37 ± 9
1.6	7H9+OADC	67 ± 7
	7H9+OADC+Tween	43 ± 15
