# Supplementary material for: Fluoromycobacteriophages for Rapid, Specific, and Sensitive Antibiotic Susceptibility Testing of Mycobacterium tuberculosis
Source: PLoS One. 2009 Mar 20;4(3):e4870. doi: 10.1371/journal.pone.0004870 (PMC2654538; doi:10.1371/journal.pone.0004870)

Figure S1: AST of a low number of *M. tuberculosis* mc^{2}6230 cells with pHAE87::*Hsp60-EGFP* and flow cytometry detection.

10^4 *M. tuberculosis* mc^{2}6230 *wt* or antibiotic resistant cells grown in the absence of Tween were infected with pHAE87::*Hsp60-EGFP* in the presence of (**A**, **E** and **J**) rifampicin or (**F**, **G** and **I**) streptomycin. Cells were fixed for 1.5 hs, spun down and resuspended in PBS prior analysis by flow cytometry. (**A**) *M. tuberculosis* mc^{2}6230 *wt* mock infected control; (**B**, **C** and **G**) *M. tuberculosis* mc^{2}6230 *wt*; (**D**, **E** and **F**) *M. tuberculosis* mc^{2}6230 Rif^R; (**H**, **I** and **J**) *M. tuberculosis* mc^{2}6230 Str^R.

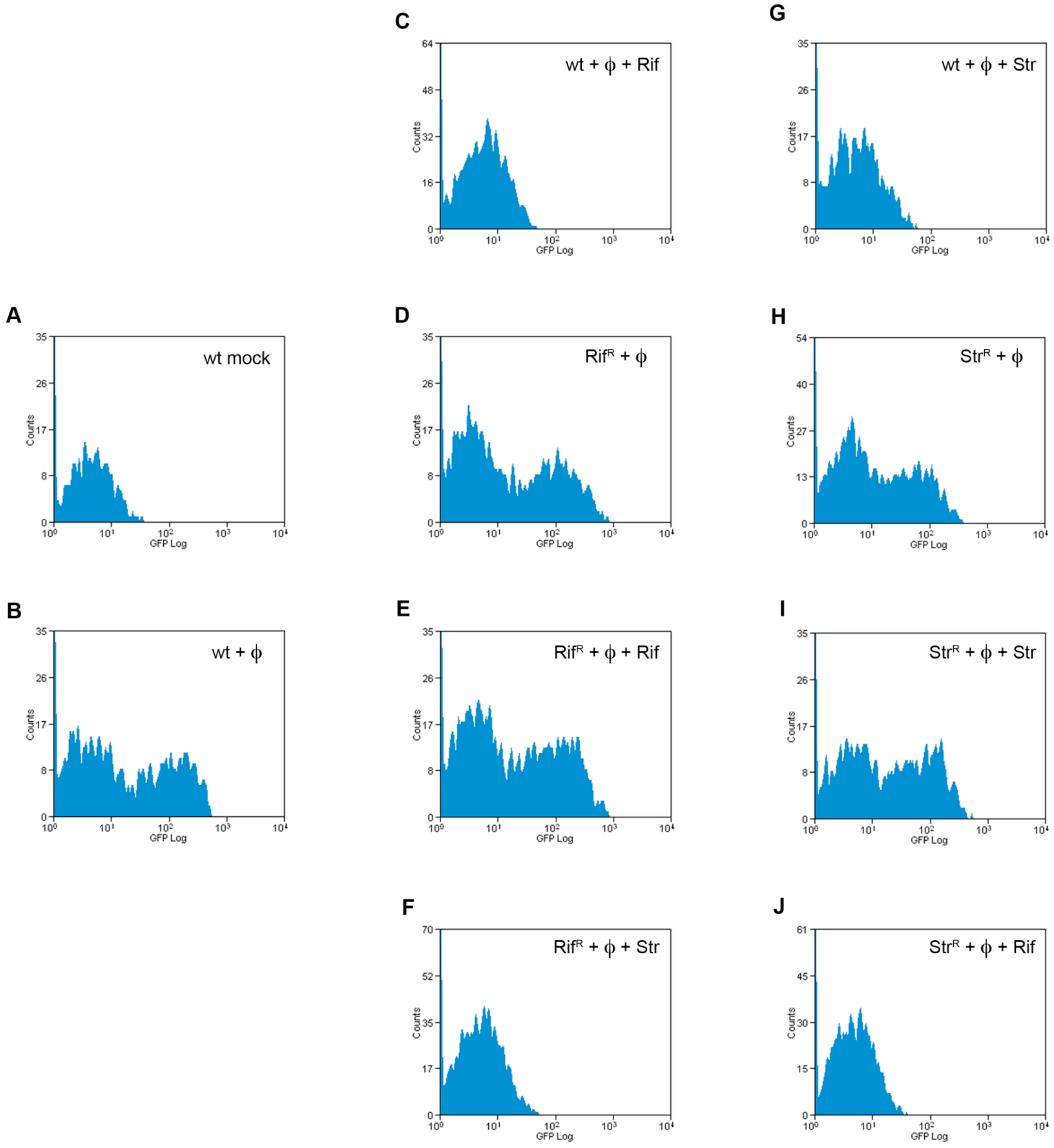

Supplement: Figure S1 — (0.31 MB PDF) [file pone.0004870.s002.pdf]
