# Supplementary material for: Fluoromycobacteriophages for Rapid, Specific, and Sensitive Antibiotic Susceptibility Testing of Mycobacterium tuberculosis
Source: PLoS One. 2009 Mar 20;4(3):e4870. doi: 10.1371/journal.pone.0004870 (PMC2654538; doi:10.1371/journal.pone.0004870)

Figure S2: Fluoromycobacteriophage *M. tuberculosis* AST using flow cytometry.

A. *M. tuberculosis* mc^{2}6230 *wt* grown in the absence of Tween detergent was infected with phAE87::*Hsp60-EGFP* in the presence of Kanamycin, fixed with paraformaldehyde, and analyzed by flow cytometr.

B. *M. tuberculosis* mc^{2}6230 strains were grown in the presence of Tween, washed and infected with phAE87::*hsp60-EGFP* in the presence of (**D**) Ofloxacin (OFL) or preincubated with (**B**) Ethambutol (EMB); (**C**) Ethionamide (ETH) or (**E**) Ofloxacin for 24 hours or (**F**) 48 hs. The intensity of fluorescence is plotted against light side scatter.In each experiment mock-infected cells are shown in red, phage infected cells in green, and antibiotic-treated phage infected cells in blue

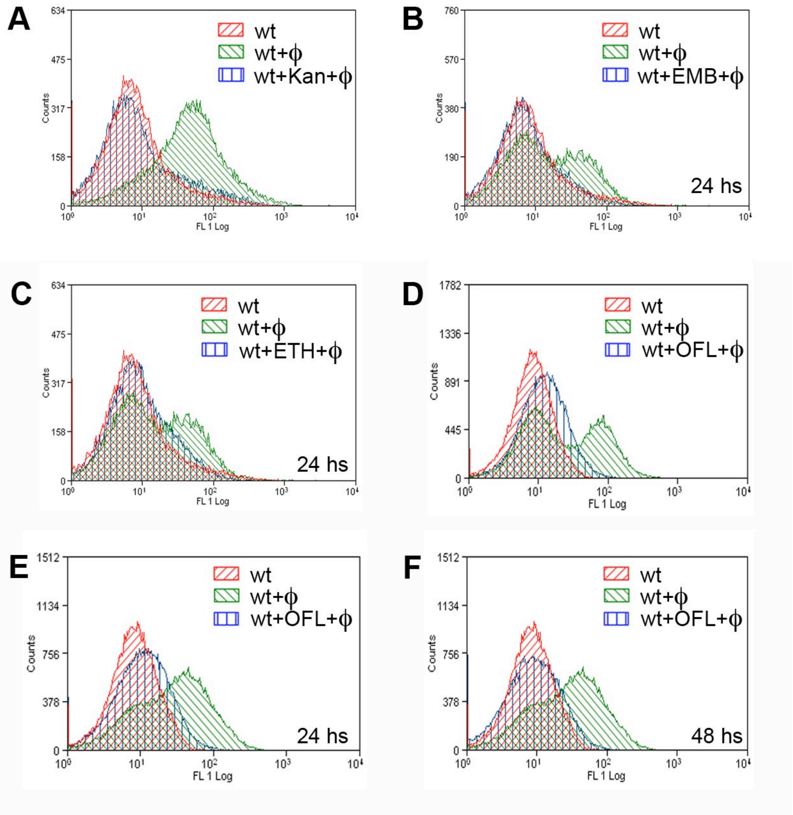

Supplement: Figure S2 — (0.76 MB PDF) [file pone.0004870.s003.pdf]
