# Supplementary material for: Fluoromycobacteriophages for Rapid, Specific, and Sensitive Antibiotic Susceptibility Testing of Mycobacterium tuberculosis
Source: PLoS One. 2009 Mar 20;4(3):e4870. doi: 10.1371/journal.pone.0004870 (PMC2654538; doi:10.1371/journal.pone.0004870)

Figure S3: Infection and AST of *M. tuberculosis* mc^{2}6230 cells with pHAE87::*Hsp60-EGFP* directly from a colony

M. tuberculosis mc^{2}6230 *wt* and antibiotic resistant cells from a resuspended colony were infected with pHAE87::*Hsp60-EGFP* in the presence of the indicated antibiotics. After infection, cells were fixed and concentrated by filtration before examination using a fluorescent microscope.

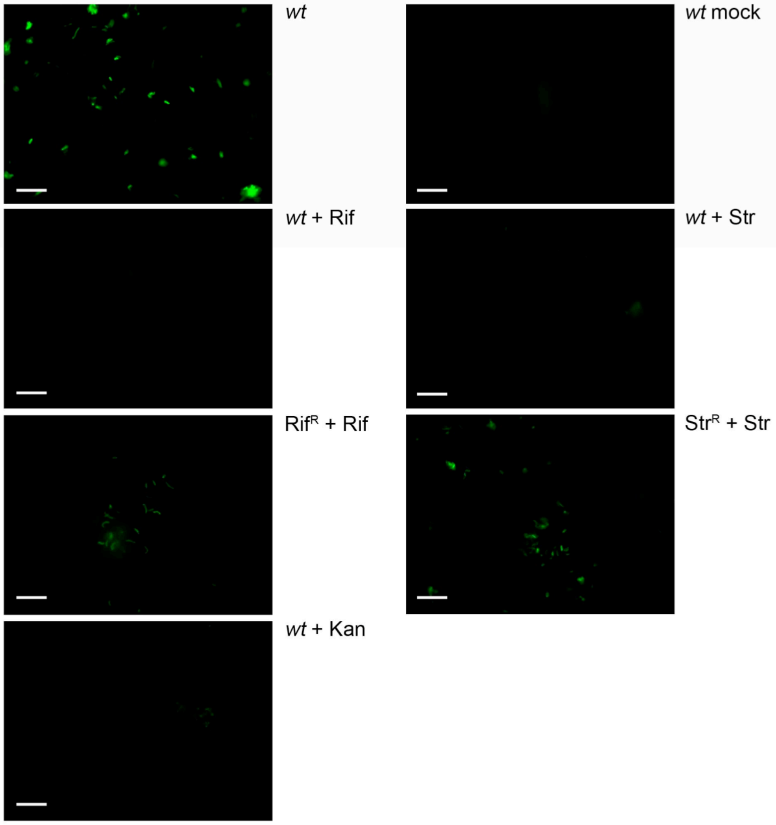

Supplement: Figure S3 — (0.17 MB PDF) [file pone.0004870.s004.pdf]
